# Candidate proteins interacting with cytoskeleton in cells from the basal airway epithelium *in vitro*

**DOI:** 10.3389/fmolb.2024.1423503

**Published:** 2024-07-30

**Authors:** Olusola A. Olatona, Sayantan R. Choudhury, Ray Kresman, Carol A. Heckman

**Affiliations:** ^1^ Department of Biological Sciences, Bowling Green State University, Bowling Green, OH, United States; ^2^ Department of Computer Science, Bowling Green State University, Bowling Green, OH, United States

**Keywords:** actin comet, intercellular junction proteins, minus-end-directed motor, keratin precursor particle, respiratory tract, tandem (hybrid) mass spectrometry, protein-protein interaction (PPI)

## Abstract

**Introduction:** The cytoskeleton consists of actin, microtubules, septins, and intermediate filaments and, in most cells, is anchored to an extracellular matrix. Each cell has a unique arrangement of this network and readjusts it from time to time. To investigate the regulation of these reorganizations, we identified interactors from extracts of four cultured lines representing basal cells from the airway epithelium.

**Methods:** After immunoprecipitation with an antibody against keratin 17, samples were processed by liquid chromatography and tandem mass spectrometry. Samples not undergoing antibody-mediated capture were processed in parallel.

**Results:** The main keratins of basal cells, namely, Krt14 (type I) and Krt5 (type II), constituted 67% of the total keratin recovered. Several other intermediate filament proteins, nestin, lamin-B1, and prelamin A/C, were present but not enriched upon immunoprecipitation. Although the class of armadillo-repeat proteins was represented by beta-catenin1 and plakoglobin, other desmosome plaque constituents were absent. Large cytolinkers were represented by the spectraplakin, microtubule-actin cross-linking factor (Macf1), which was enriched by immunoprecipitation, and the plakin, plectin, which was not enriched. Subunits of actin filaments and microtubules, along with numerous proteins associated with them, were recovered in both immunoprecipitated samples and those lacking the capture step. Coefficients of determination were computed based on abundance. The actin-associated proteins, alpha-spectrin and brain-specific angiogenesis inhibitor (Baiaip2l), were modestly correlated with keratin abundance but highly correlated with one another and with the keratin-binding protein, annexin A2. This interaction network resembled the pedestal formed by pathogenic *Escherichia coli*. Microtubule-associated proteins, dynamin 1-like protein and cytoplasmic dynein 1 heavy chain (Dync1h1), were enriched by immunoprecipitation, suggesting association with keratins, whereas kinesin-1 heavy chain and microtubule-associated protein retinitis pigmentosa 1 (EB1), were not enriched. Dync1h1 abundance was negatively correlated with that of all the septins, suggesting resemblance to a known antagonistic septin-dynein 1 relationship on microtubules.

**Conclusion:** The cell lines showed remarkable uniformity with respect to the candidates interacting with cytoskeleton. The alpha-spectrin-Baiap2l network may link actin filaments to keratin precursor particles. A smaller interaction network centered on Dync1h1 was negatively correlated with all spectrin-Baiap2l constituents, suggesting that it and its binding partners are excluded from the pedestal-like domain.

## 1 Introduction

Along with a reduced ability to adhere to substrata, structural softening is a nearly universal characteristics of cancer cells. In previous studies, we correlated such structural changes with alterations in various biophysical traits (Heckman et al., 1987; Heckman and Jamasbi, 1999). Several review articles on this subject argue that the changes are related to the deregulation of growth control in the cancer cell [see for reviews (Alibert et al., 2017; Sheetz, 2019; Xin et al., 2023)]. Experimental studies on the human breast cancer cell lines, MDA MB231 and MCF10A, suggest that reduced adhesion may result from altered gene expression. Although there was little difference in the content of proteins such as paxillin, focal adhesion kinase, or integrins, the adhesive strength of the nontumorigenic MCF10A cells was an estimated 10-fold greater than the malignant MDA MB231 cells in the absence of cations (Fuhrmann et al., 2017). Studies of different populations, selected for lower or higher adhesiveness, found that 500 genes were differentially expressed. Several, KIF14, DYNC1H1, and AKAP9, were associated with microtubules. The list also included growth arrest specific 2 like 3 (GAS2L3), a cell cycle regulatory protein that links microtubules and actin filaments (Beri et al., 2020). Other cross-linkers, including KN motif and ankyrin repeat domains (Li et al., 2023) and an ortholog of Macf1, Short stop (Zhao et al., 2022), were thought to coordinate force transmission with microtubule recruitment. A few differentially expressed proteins may be able to modify the composition or rate of turnover of focal adhesions. This introduces a dilemma, because the cytoskeleton is integrated with adhesions by connections among proteins, making up a gigantic network. To address this problem, a database of putative interactors with cytoskeleton is needed.

We previously found that malignant respiratory airway cells were characterized by an aberrant arrangement of keratins (Manger and Heckman, 1982). The pattern of keratin rearrangement was intuitively recognizable. This would rarely be the case for rearrangement of the networks of actin or microtubules. Although the latter networks are studied more frequently than the keratins, they are known to interact with keratins in several ways. Keratin precursor particles of keratinocytes interact with the actin filaments that maintain membrane tension in the lamellipodium (Pora et al., 2020). Thus, the rearrangement observed could depend on the membrane force, and changes in either or both of these cytoskeletal networks may cause softening and/or reduced adhesiveness. In previous studies of the airway cells, actin was recovered in the pellet after extraction of the soluble proteins. It could not be dissociated even by high concentrations of salts and a nonionic detergent (Blobel et al., 1984; Heckman and Mayumi, 2022). This suggests tight binding between the two proteins, consistent with the idea that keratin rearrangement in malignant cells could depend on the membrane force maintained by actin. Previous work also disclosed that long keratin fibrils, like other types of intermediate filaments, undergo anterograde transport on microtubules, mediated by kinesin-1 (Robert et al., 2019).

Whereas the architectural elements have been known for decades, it has now become clear that they form networks. There are numerous links among the networks that may join them in the same way architectural elements, such as joists and beams, knit structures together in a building. Neither the cross-linkers nor the scaffolds that perform the joining functions are well-known. If these proteins can be identified, their roles can be tested to determine whether any can account for the abnormal traits. Putative scaffold interactors and cytoskeletal cross-linkers were found here by comparing the proteins in four lines representing basal cells of the respiratory airway epithelium. It was assumed that the linkers and scaffolds are characteristic of basal cells. Thus, a list of proteins common to all lines will include them. Using tandem mass spectrometry to analyze the proteins, we found subsets associated with actin and microtubules. The keratin-associated proteins are less understood and were grouped with scaffolds and scaffold interactors. This novel method yielded a database of candidates that may link the structural elements. The results suggest that proteins whose abundance is correlated represent networks of interacting constituents. If they form hetero-multimeric clusters, cooperative interactions among the proteins may be required to stabilize the structures. Further work is needed to confirm the existence of each complex and discern its role and localization in cells.

## 2 Materials and methods

### 2.1 Cell lines and cell culture

Cell lines were derived from the upper airway epithelium of pathogen-free Fischer 344 inbred rats. The BP3 line was derived from tumors induced with benzo(a)pyrene and serially transplanted in immunocompetent F344 rats (Jamasbi et al., 1978; Heckman and Olson, 1979). It was malignant upon injection of as few as 100 cells and readily metastasized to distant sites (Heckman and Olson, 1979; Heckman and Mayumi, 2022). The 1000 W line was derived from tracheal epithelium pre-exposed to 7,12-dimethylbenz(a)anthracene (Marchok et al., 1978). For comparison, the 2C1 and 4C9 lines were derived from rat tracheal organ explants treated with phorbol 12-myristate 13-acetate (Steele et al., 1978). The 2C1 line was not tumorigenic. The 4C9 line became tumorigenic after prolonged serial passage in culture and did not metastasize in host animals (Manger and Heckman, 1982). Ethical approval was not required for the studies involving animals, because the cell lines were provided by other researchers. All lines were grown in Waymouth MB 752/1 medium (Sigma-Aldrich, Burlington, MA) supplemented with 10% fetal bovine serum, 0.1 μg/mL hydrocortisone, and 0.1 μg/mL insulin. Cells were plated at 7–12 × 10^5^ per 100 mm dish and maintained at 37°C in a humidified atmosphere of 5% CO_2_. Cells were passaged using Ca^2+^- and Mg^2+^-free solution containing ethylene diamine tetraacetate (EDTA) and trypsin (Life Science Technologies, Grand Island, NY).

### 2.2 Protein extraction and Lowry assay

All chemicals were from Sigma-Aldrich unless otherwise designated. To prepare cytoskeletons, cell cultures were rinsed thrice with cytoskeletal buffer [140 mM NaCl (Fisher Scientific, Fair Lawn, NJ), 5 mM MgCl_2_, (Mallinckrodt, St. Louis, MO), 20 mM tris (hydroxymethyl)aminomethane (TRIS), pH 7.6] and exposed to radioimmunoprecipitation assay (RIPA) solution. RIPA, containing 1% Triton X-100 (TX-100), 0.1% sodium deoxycholate, 0.1% sodium dodecylsulphate (SDS), 20 mM TRIS (pH 8.0), 1 mM EDTA (Fisher Scientific), 1 mM ethylene glycol tetraacetate, 140 mM NaCl, and 1 mM phenylmethylsulfonylfluoride (PMSF), heated to 90°C, was added to the culture. Cells were removed with a rubber policeman, and samples were flash-frozen and kept at −196°C until processing. Upon thawing, the protein concentrations were estimated using the Folin-Lowry method (Lowry et al., 1951).

The extracts made with the RIPA lysis mixture were compared to keratin-rich fractions isolated from respiratory airway cells. The latter samples were made by rinsing the dishes twice with isotonic TRIS buffer, followed by 1% TX-100 for 4 min. The cells were harvested with a rubber policeman and the cytoskeleton was pelleted by centrifugation at l, 500 × g at 4°C. Pellets were resuspended in 2% SDS, 10% glycerol (Fisher Scientific), 5% β-mercaptoethanol (Fisher Scientific), and 1 mM PMSF (pH 8.3), boiled until solubilized, and then analyzed as previously described (Heckman and Mayumi, 2022).

### 2.3 Antibody generation and immunoprecipitation (IP)

The Krt14 and Krt17 spots were excised from two-dimensional gels of keratins, prepared as described above (see Section 2.2). The combined spots were injected into a rabbit by the intradermal route, followed by booster doses, and the antiserum was collected after the last injection. To test antibody specificity, spots representing Krt14 and Krt17 were ground in a mortar and pestle with 300 mL 4% SDS, 150 mM TRIS, and 3 mM dithiothreitol. Serial dilutions of the material were made with TRIS buffer, and 2 mL aliquots were placed on Immobilon membranes. The blots were treated with 1:500 dilution of anti-keratin antibody, followed by 1:250 goat anti-rabbit horseradish peroxidase-tagged antibody (Millipore Corporation, Temecula, CA). They were developed in 2.5 mM 3,3′-diaminobenzidine in TRIS buffer with 0.03% H_2_O_2_ (Macron, Center Valley, PA), as described previously (Heckman et al., 2017). The antibody proved to be specific for Krt17 (Supplementary Material S1).

IP was done by adding 70 µL of lysate into an Eppendorf tube containing 400 µL of deionized water and 500 µL 2X concentrate of IP buffer at pH 7.4 [2% TX-100, 300 mM NaCl, 20 mM TRIS, 2 mM EDTA, 2 mM ethylene glycol tetraacetate, 0.4 mM sodium orthovanadate, and 1.0% Nonidet P-40 (BDH Chemicals, Poole, United Kingdom), with PMSF added to a final concentration of 1 mM]. An aliquot of 16 µL anti-keratin in 50% glycerol was added. Mixtures without antibody served as the controls for nonspecific binding. Tubes were mixed by inversion and incubated at 4°C with rotation for an hour. An aliquot of 100 µL 10% protein-A agarose beads (Life Technologies, Gaithersburg, MD) was added to each tube, and the tubes were incubated for an hour. Beads were recovered by centrifuging at 9,000 rpm at 4°C and rinsing twice with IP buffer. Upon recovery, they were suspended in phosphate-buffered saline and sent to Poochon Scientific, Frederick, MD, for mass spectrometry analysis.

### 2.4 Liquid chromatography and mass spectrometry-mass spectrometry (LC-MS-MS)

Samples were treated by in-solution digestion with trypsin/lys-C protease mix. Proteins were reduced with dithiothreitol at 56°C for 45 min followed by alkylation with iodoacetamide for 30 min at room temperature in the dark. Alkylated proteins were precipitated by 80% acetone followed by trypsin/lysC digestion at 37°C for 16 h. The peptide mixture was then concentrated and desalted using C18 Zip-tip. The peptides were made up in 20 µL of 0.1% formic acid and a 12 µL aliquot was analyzed by 60 min LC-MS-MS run. Mixtures were loaded onto a peptide trap cartridge at a flow rate of 5 μL/min. Trapped peptides were eluted onto a reversed-phase Easy-Spray Column PepMap RSLC, C18, 2 μM, 100A, 75 μm × 250 mm (Thermo Scientific) using a gradient of acetonitrile (3%–36%) in 0.1% formic acid. Peptides were eluted from the column, ionized, and sprayed into the spectrometer, using a Nano Easy-Spray Ion Source (Thermo). Settings were: spray voltage, 1.6 kV, capillary temperature, 275°C. The 15 most intense multiply charged ions (z ≥ 2) were sequentially isolated and fragmented in the octopole collision cell by high energy collisional dissociation (HCD) using normalized HCD collision energy 30 with automatic gain control target 1 × 10^5^ and maximum injection time of 200 ms at 17,500 resolution. The isolation window was set to 1.6. Dynamic exclusion was set to 20 s. Charge state screening was enabled to reject unassigned and 1+ and 7+ or higher charge states ions.

Raw data files obtained from the analysis were processed using Proteome Discoverer 2.4 software (ThermoFisher, San Jose, CA) based on the SEQUEST algorithm. Reports included data on protein assemblies, peptide spectrum matches, and MS1 peak area. MS profile searches were performed against the rat sequence database. Searches of immunoglobulin sequences were performed against the rabbit database. Cysteine carbamidomethylation (+57.021 Da) was set as a fixed modification. Oxidation (M), deamidation (N, Q), acetylation (K), phosphorylation (S, T, Y), HexNAc (S, T, N), and ubiquitin-K (K) (+114.043 Da) were designated as dynamic modifications. Minimum peptide length was five amino acids. Precursor mass and the fragment mass tolerance were set to 15 ppm and 0.05 Da, respectively. A maximum false discovery rate (FDR) of 0.01 was applied.

### 2.5 Database refinement

To assemble the database, we selected proteins with appropriate subcellular localizations, as determined from the UniProtKB (www.uniprot.org/uniprot) and Human Protein Atlas sites (www.proteinatlas.org). Searches were performed using the accession numbers or the gene name and organism “*Rattus norvegicus*.” Locations, e.g., cell junctions, endosomes, plasma membrane, vesicles, or cell periphery, which could attach scaffold proteins, were included. The resulting protein lists had 867 (4C9), 669 (2C1), 898 (1,000 W), and 813 (BP3) entries. The threshold required for further analysis was a posterior error probability (PEP) of <0.01. PEP is a measure of the probability that a given spectrum match is incorrect. The proteins were not considered further if the q-value was >0, reflecting overall quality of the discrimination procedure based on the false discovery rate for that sample. Proteins were considered further if the number of unique peptides was ≥2. With these criteria, the number of candidates identified per sample were: 362 (4C9), 244 (2C1), 355 (1,000 W), and 262 (BP3).

### 2.6 Statistical analysis

**FIGURE 1:**
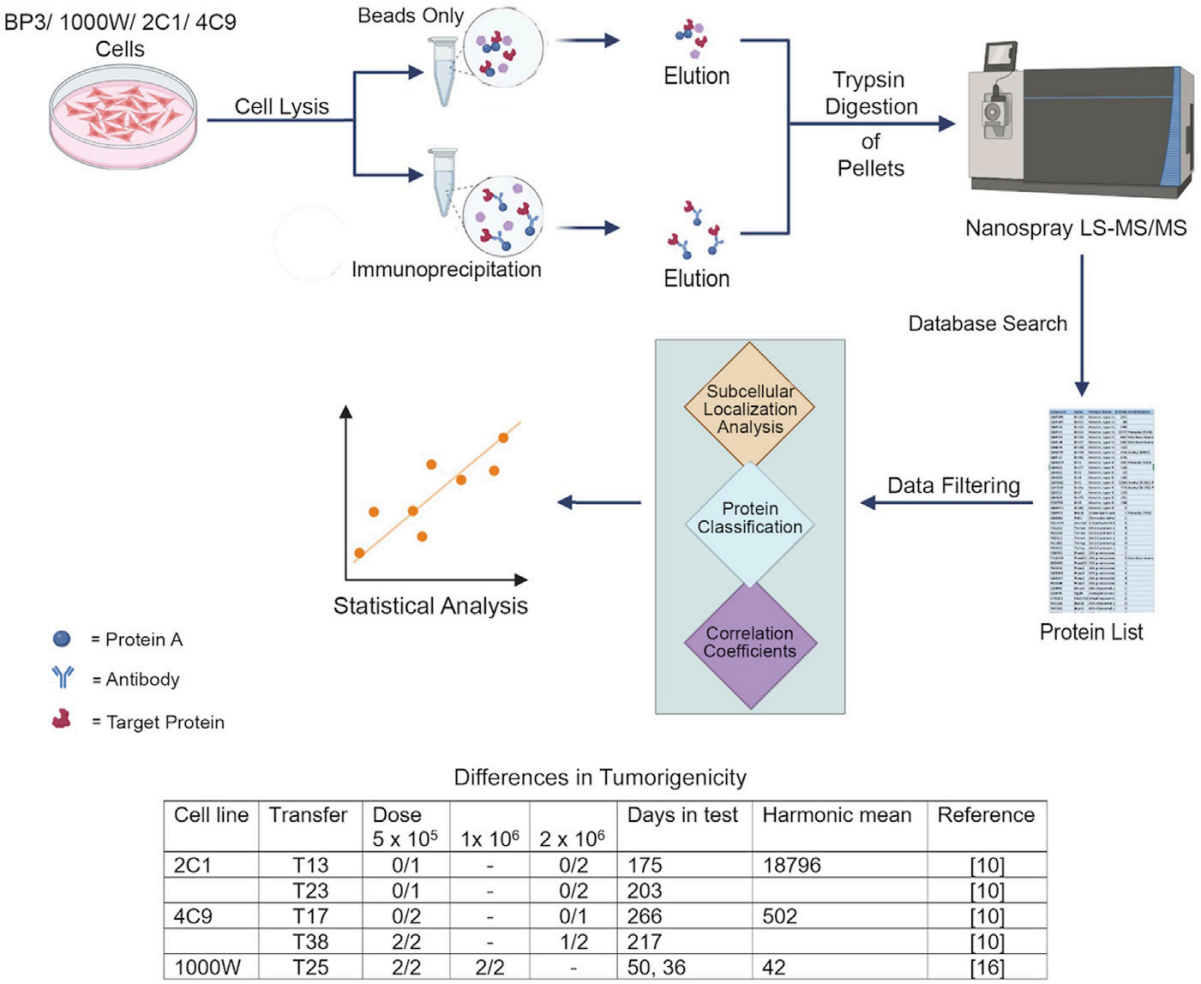
Workflow of the analysis. Three of cell lines were nontumorigenic or moderately tumorigenic. One cell line, BP3, was highly malignant and caused tumors at a dose of 100 cells (Heckman and Olson, 1979; Heckman and Mayumi, 2022).

Proteins showing at least a two-fold increase in abundance due to capture by IP, relative to samples recovered at the same time without capture, were identified. The Mann-Whitney U test was used, because the data did not meet the normality assumption required for a *t*-test. Proteins with a total abundance of 9E+06, when summed over all samples, were further evaluated. Cytoskeletal constituents, actin and tubulin, were analyzed together with their associated proteins, as were constituents that could potentially interact with the keratins or other structures, which are specified above. Proteins with an unknown localization were analyzed if they could interact with scaffolds. The remaining proteins, not enriched due to capture by IP, were analyzed separately. The Pearson correlation coefficients were determined to learn whether the proteins were correlated with keratin content. Keratin mass was calculated as a fraction of the abundance of all proteins, i.e. 0.5464 and 0.3468 for two samples without antibody, and 0.3474 (4C9), 0.4903 (2C1), 0.5540 (1,000 W), and 0.6243 (BP3) for the respective lines. If the protein of interest was missing from one sample, the data from that sample were not included in the analysis. Values were determined using Social Science Statistics at https:// www.socscistatistics.com/tests/pearson/default2.aspx. For proteins missing from two samples, Pearson correlation coefficients could not be determined. Unless R values are specified in the following text, the coefficient of determination (CD) is presented to enable the proteins to be evaluated in relation to overall keratin content. The experimental design is illustrated in Figure 1.

### 2.7 Computational methods

The Python Anaconda environment (Anaconda, 2020), a software paradigm for data science and life sciences, was employed to calculate CDs for protein abundance. A data pipeline was developed as a Jupyter Notebook application. This consisted of data clean up, validation, and processing, and the data were stored in a csv file with one column/protein. Data were read into a Pandas DataFrame and, as is customary, missing values were replaced by NaN. Every pairwise comparison was validated to ensure that at least 5 values were non-null values, using the dropna method with a threshold of 2. Consistent with this threshold, 1,008 pairs, out of a total of 19,110 pairs, were excluded from further analysis. Python’s SciPy library was employed in the processing step to perform Pearson correlation. Its method, pearsonr, returns a dictionary object with a key value pair. Key is set to the name of the protein pair and value gives rise to a list that includes the CD. The dictionary object was converted to a DataFrame and the result stored as a csv file.

## 3 Results

The keratins recovered were typical of those found in basal cells. Krt14 and Krt5 were the main type I and type II keratins in basal cells, respectively, and they made up 67% of the total keratin content in the samples analyzed here. Krt17 and Krt6A together accounted for another 21%. Typical proteins of differentiated epidermal cells, e.g., Krt1-4 and Krt10, were poorly represented. Krt1-4 made up 3% and Krt10 1% of the total. Details are given elsewhere (Olatona, 2023). Two armadillo-repeat proteins, β-catenin and plakoglobin, were found, but plakophilin, desmocollin, and desmoglein, were absent, as was the keratin-binding protein, desmoplakin. This suggested that the junctions of respiratory airway cells had a simpler structure than the desmosomes between epidermal cells.

### 3.1 Proteins selectively recovered with anti-keratin antibody

**FIGURE 2:**
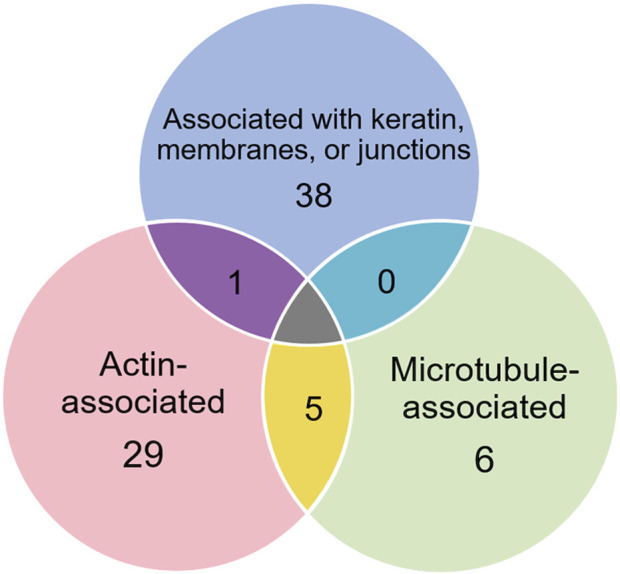
Venn diagram showing different classes of proteins in preparations enriched by IP with anti-keratin antibody, identifying those that cross-link different cytoskeletal elements.

Of the proteins known to interact with one of the main architectural elements, there were 29 associated with actin, including β-actin itself, six with microtubules, including two tubulin subunits, and 38 proteins that bind to keratins, membranes, cell-cell junctions, intracellular vesicles, or cell-substrate adhesions. We identified a total of six cross-linking proteins among this group. These cross-links are highlighted in a Venn diagram (Figure 2) and in Tables 1, 2. Several proteins enriched by IP were found in cytoskeletal pellets from cells treated by TX-100 extraction. Whereas actin-related protein 3, annexin A2, and microtubule-actin cross-linking factor were in Krt14, Krt19, and Krt5 spots, respectively, β-actin was present in all spots analyzed from two-dimensional gels. A microtubule-associated protein, Dynamin-1-like, was found by IP (Table 2) and in the Krt17 spot (see Supplementary Material S6). It showed no correlation with keratin or Krt17 abundance, however. The only keratin-associated protein found within a keratin spot and also enriched by IP after RIPA was glutamine gamma-glutamyl transferase (Tgm1). It has a role in keratin structure as discussed below (see Section 3.1.3). Of the soluble proteins, 26S proteasome regulatory subunit 4 (Psmc1) and T-complex protein 1 subunit delta (Cct4) were found with Krt5 (see Supplementary Material S6).

**TABLE 1:** Actin-associated proteins selectively recovered with antibody.

Accession	Gene	Protein description	MW	R^2^	Modifications
P60711	Actb	Actin, cytoplasmic 1	41.7	0.312	Met-loss + Acetyl [N-Term]
O88656	Arpc1b	Actin-related protein 2/3 complex subunit 1B	41.0	0.015	
Q5M7U6	Actr2	Actin-related protein 2	44.7	0.016	
Q4V7C7	Actr3	Actin-related protein 3	47.3	0.066	
O35889	Afdn	Afadin	207.5	0.002	
*P07150*	*Anxa1* ^a^	*Annexin A1*	*22.4*	*0.008*	*Met-loss + Acetyl [N-Term]*
**Q07936**	**Anxa2^a^**	**Annexin A2**	**38.7**	**0.364**	**Met-loss + Acetyl [N-Term]**
Q08163	Cap1	Adenylyl cyclase-associated protein 1	51.6	0.021	Met-loss + Acetyl [N-Term]
Q9Z1P2	Actn1	Alpha-actinin-1	102.9	0.057	
Q6GMN2	Baiap2	Brain-specific angiogenesis inhibitor 1-associated protein 2	59.1	0.067	
Q3KR97	Baiap2l1	Brain-specific angiogenesis inhibitor-1-associated protein 2-like protein 1	57.4	0.578	
O89046	Coro1b	Coronin-1B	53.8	0.070	
Q5XI32	Capzb	F-actin-capping protein subunit beta	30.6	0.124	
P31977	Ezr	Ezrin	69.3	0.001	
P85845	Fscn1	Fascin	54.5	0.448	
D3ZHA0	Flnc	Filamin-C	290.8	0.297	
Q68FP1	Gsn	Gelsolin	86	0.326	
F1LR10	Lima1	LIM domain and actin-binding protein 1	83.7	0.410	Phospho [S360; S488]
*D3ZHV2*	*Macf1* ^a^	*Microtubule-actin cross-linking factor 1*	*619.2*	*0.376*	
Q64119	Myl6	Myosin light polypeptide 6	17	0.091	Met-loss + Acetyl [N-Term]
P18666	Myl12b	Myosin regulatory light chain 12B	19.8	0.358	
Q62812	Myh9	Myosin-9	226.2	0.004	Phospho [S1944]; HexNAc [S1678]
Q62920	Pdlim5	PDZ and LIM domain protein 5	63.2	0.543	
*Q9WVC0*	*Septin7* ^a^	*Septin-7*	*50.5*	*0.520*	
P16086	Sptan1	Spectrin alpha chain, non-erythrocytic 1	284.5	0.788	
Q63610	Tpm3	Tropomyosin alpha-3 chain	29.0	0.392	Met-loss + Acetyl [N-Term]
Q05096	Myo1b	Unconventional myosin-Ib	131.8	0.104	
Q63357	Myo1d	Unconventional myosin-Id	116.0	0.049	Met-loss + Acetyl [N-Term]; Phospho [Y118]
Q5RKI0	Wdr1	WD repeat-containing protein 1/actin-interacting protein 1 (AIP1)	66.1	0.029	

^a^ Legend: *Italics* represent proteins associated with both actin and microtubules; **Bold** represents proteins associated with actin and keratin.

**TABLE 2:** Microtubule-associated proteins selectively recovered with antibody.

Accession	Gene name	Protein description	MW	R^2^	Modifications
P38650	Dync1h1	Cytoplasmic dynein 1 heavy chain 1	531.9	0.716	
O35303	Dnm1l	Dynamin-1-like protein	83.9	0.088	
*Q91Y81*	*Septin2*	*Septin-2*	*41.6*	*0.501*	
*Q9QZR6*	*Septin9*	*Septin-9*	*63.8*	*0.656*	
P68370/Q6P9V9	Tuba1a	Tubulin alpha-1A (with 1B)	50.1	0.303	
Q5XIF6	Tuba4a	Tubulin alpha-4A chain	49.9	0.007	

Legend: *Italics* represent proteins associated with microtubules and actin.

#### 3.1.1 Actin-associated proteins

**FIGURE 3:**
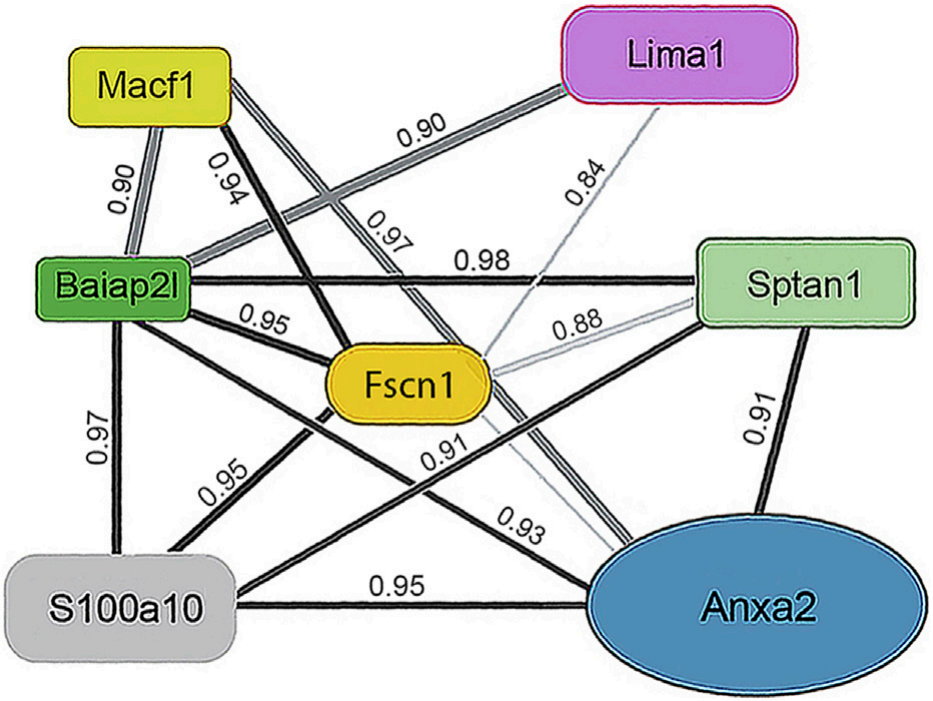
PPIN1 resembles the spectrin-IRSp53 pedestal. CDs are shown on the edges, and protein abundance values are given in Supplementary Material S5. Within the core region, the putative interactors’ edges numbered four (Baiap2l) or three (Anxa2) indicating their connectedness in the network. Additional proteins binding Anxa2, but not shown on this diagram, included Myl12b (CD 0.95), Tpm3 (CD 0.93), and Lima1 itself (CD 0.92). The presence of tropomyosin and myosin light chain on the PPIN suggests they stabilize an arrangement of actin filaments.

One subunit of the actin filament, β-actin, and 28 actin accessories were selectively recovered by IP. The abundance of actin subunit, Actb, was positively correlated with myosin light chain 12B (Myl12b, CD 0.93) and a nuclear protein, lamin B1 (Lmnb1, CD 0.90). Elevated CDs between actin and proteins of Table 1 were limited to Lima1, Anxa2, Macf1, and Tpm3, and ranged from 0.82 to 0.87 (see Supplementary Material S5). As shown below in Table 1, α-spectrin (Sptan1) showed the highest CD with keratin abundance, and its CDs with two other actin-associated proteins, Baiap2l and Anxa2, were also high (Figure 3). A Baiap2 isoform, IRSp53, was known to form a complex with spectrin and initiate actin polymerization in pedestals formed by the virulent pathogen, enterohaemorrhagic *Escherichia coli* (Ruetz et al., 2012). Whereas the α-spectrin-Baiap2l relationship suggested that a similar complex was formed, no WH2 domain-containing protein was found here. Instead, the complex included Anxa2. A pleiotropic calcium- and anionic phospholipid-binding protein, Anxa2 was the sole protein of Table 1 known to bind to intermediate filaments (Chung et al., 2012; Chang et al., 2018), as highlighted in bold typeface. Although Anxa2 formed a heterotetramer with S100a10 (Bharadwaj et al., 2013), S100a10 was not specifically enriched by IP in these samples and was probably captured together with other proteins of an α-spectrin-Baiap2l-fascin (Fscn1) scaffold (Figure 3, see Section 3.2). In addition to Fscn1, which was correlated with Baiap2l, Macf1 and Lima1 were additional Baiap2l binding partners (Figure 3). The data suggested that Baiap2l and Sptan1 bind Anxa2, and then bind keratins using Anxa2 as an intermediary.

Because the core of Figure 3 resembled a pedestal-like network based on Baiap2l, it is called PPIN1. Baiap2l had high CDs with the other four proteins in the core, and all the Pearson correlations indicated a positive relationship. Fscn1 was strongly correlated with only two but was weakly correlated with Anxa2. It also had correlations with the actin-associated proteins, Lima1 and Macf1 (Figure 3). The many high, positive values suggested cooperative assembly of actin-associated proteins. Thus, it was surprising to find that these Fscn1 partners were not associated with Fscn1 in the STRING database (Supplementary Material S2).

Evaluation of the STRING database (Szklarczyk et al., 2015) showed no representation of Sptan1 or Anxa2 binding to Baiap2l (see Supplementary Materials S3, S4). Moreover, when we analyzed the keratins in pellets after extraction of the soluble proteins, the only constituent of the five-member PPIN1 still bound to keratin was Anxa2. However, two actin-binding proteins were found, namely, actin-related protein 3 and Macf1 (see Supplementary Material S6). Macf1 is related to three proteins within PPIN1 but more distantly than fascin, Anxa2, or S100a10.

Although the abundance of Krt17 in the samples was low, its values were tested for correlation with other proteins. The pairwise CD for Baiap2-Krt17 was 0.89. Baiap2 was an alternative IRSp53 isoform, which showed no correlation with total keratins (Table 1). That it may selectively bind Krt17 was supported by the observation of correlations between Baiap2 and known keratin-binding proteins, 14-3-3 beta/alpha (Ywhab, 0.90) and 14-3-3 eta (Ywhah, 0.88). Only 14-3-3 beta/alpha was correlated with Krt17 (CD 0.86). However, Krt17 may use weak relationships with adapters, including 14-3-3 alpha/beta and Anxa5 (CD 0.85), to bind to other cytoskeletal structures.

Several actin-associated proteins recovered by IP were known to process monomers or mediate filament disassembly or severing. Cap1 recharges actin monomers with ATP and facilitates their subsequent binding to profilin (Bertling et al., 2004; Colin et al., 2023). Wdr1 and Coro1b cooperate with actin depolymerization factors in disassembly (Tang et al., 2020). Gelsolin severs filaments and caps the minus ends. Another actin accessory, Capzb, functions as a barbed-end cap. A survey of interactions among these proteins suggested that Wdr1 and Cap1 (CD 0.95) had the greatest pairwise interaction.

#### 3.1.2 Microtubule-associated proteins

Among the candidates evaluated, six were associated with microtubules, including α-tubulin subunits, 1 and 4. As in the case of actin subunits, the tubulin subunits had low CDs with other proteins of Table 2 (see Supplementary Material S5). Likewise, the α-tubulins showed no CDs with β-tubulin subunits above 0.69, suggesting that the whole microtubule was partly disassembled into subunits. Cytoplasmic dynein 1 heavy chain (Dync1h1) and septin-9 showed weak CDs with total keratin content (Table 2). The Pearson correlation coefficients, R, had opposite signs, however. This suggested that septin-9 was concentrated in keratin-rich samples, while Dync1h was depleted. Of the septins, septin-10 was present in too few samples to be analyzed, but septins −2 and −7 had weak interactions with overall keratin content (Tables 1–3). Filament formation by septins was well-known (Cavini et al., 2021). Our analysis suggested that this association was maintained in RIPA lysates, as the content of each was correlated with that of the other septins (R of 0.69–0.96). Thus, it was probable that septins work cooperatively to interact with other proteins.

**TABLE 3:** Proteins associated with keratins or other structures selectively recovered with antibody.

Accession	Gene	Protein description	MW	R^2^	Modifications
P35213	Ywhab	14-3-3 protein beta/alpha	28.2	0.015	
P62260	Ywhae	14-3-3 protein epsilon	29.2	0.081	
P68511	Ywhah	14-3-3 protein eta	28.2	0.001	Met-loss + Acetyl [N-Term]
P61983	Ywhag	14-3-3 protein gamma	41.9	0.001	
P04764	Eno1	Alpha-enolase	47.1	0.396	
P14669	Anxa3	Annexin A3	49.6	0.016	
P55260	Anxa4	Annexin A4	35.8	0.088	
P14668	Anxa5	Annexin A5	35.7	0.028	
Q9R1T1	Banf1	Barrier-to-autointegration factor	10	0.316	
Q9WTT7	Bzw2	Basic leucine zipper and W2 domain-containing protein 2 (with Bzw1)	48	0.002	
Q9WU82	Ctnnb1	Catenin beta-1	85.4	0.209	
Q8CFN2	Cdc42	Cell division control protein 42 homolog	21.2	0.141	
Q63532	Sprr1a	Cornifin-A	16.7	0.842	
Q5M9G3	Caprin1	Cytoplasmic activation/proliferation-associated protein-1	78.1	0.580	
Q7TQ20	Dnajc2	DnaJ homolog subfamily C member 2	71.7	0.577	
Q62940	Nedd4	E3 ubiquitin-protein ligase NEDD4	102.3	0.005	
Q641Z6	Ehd1	EH domain-containing protein 1 (with Ehd2)	60.6	0.000	
P68101	Eif2s1	Eukaryotic translation initiation factor 2 subunit 1	36.1	0.044	
Q4G061	Eif3b	Eukaryotic translation initiation factor 3 subunit B	90.9	0.451	Acetyl [N-Term]
Q6AYK8	Eif3d	Eukaryotic translation initiation factor 3 subunit D	63.9	0.154	
Q641X8	Eif3e	Eukaryotic translation initiation factor 3 subunit E	52.2	0.019	Met-loss + Acetyl [N-Term]
Q5RK09	Eif3g	Eukaryotic translation initiation factor 3 subunit G	35.6	0.000	
B2GUV7	Eif5b	Eukaryotic translation initiation factor 5B	137.6	0.012	
P08699	Lgals3	Galectin-3	27.2	0.072	
P04897	Gnai2	Guanine nucleotide-binding protein G(i) subunit alpha-2	40.5	0.109	
P06761	Hspa5	Endoplasmic reticulum chaperone BiP	72.3	0.101	
P42930	Hspb1	Heat shock protein beta-1	22.9	0.449	
P82995	Hsp90aa1	Heat shock protein HSP 90-alpha	84.8	0.385	Phospho [S231]
P34058	Hsp90ab1	Heat shock protein HSP 90-beta	83.2	0.355	Phospho [S255]
Q64632	Itgb4	Integrin beta-4	200.5	0.390	
Q7TQM5	Kprp	Keratinocyte proline-rich protein	76.3	0.347	
P19804	Nme2	Nucleoside diphosphate kinase B	17.3	0.070	
P23606	Tgm1	Protein-glutamine gamma-glutamyltransferase K	90.7	0.398	
B4F7E8	Niban2	Protein Niban 2	84.7	0.081	
P50399	Gdi2	Rab GDP dissociation inhibitor beta	50.5	0.351	
Q6RUV5	Rac1	Ras-related C3 botulinum toxin substrate 1	21.4	0.028	
B3GNI6	Septin11	Septin-11	49.7	0.268	Met-loss + Acetyl [N-Term]
Q2LAP6	Tes	Testin	47.6	0.011	

**FIGURE 4:**
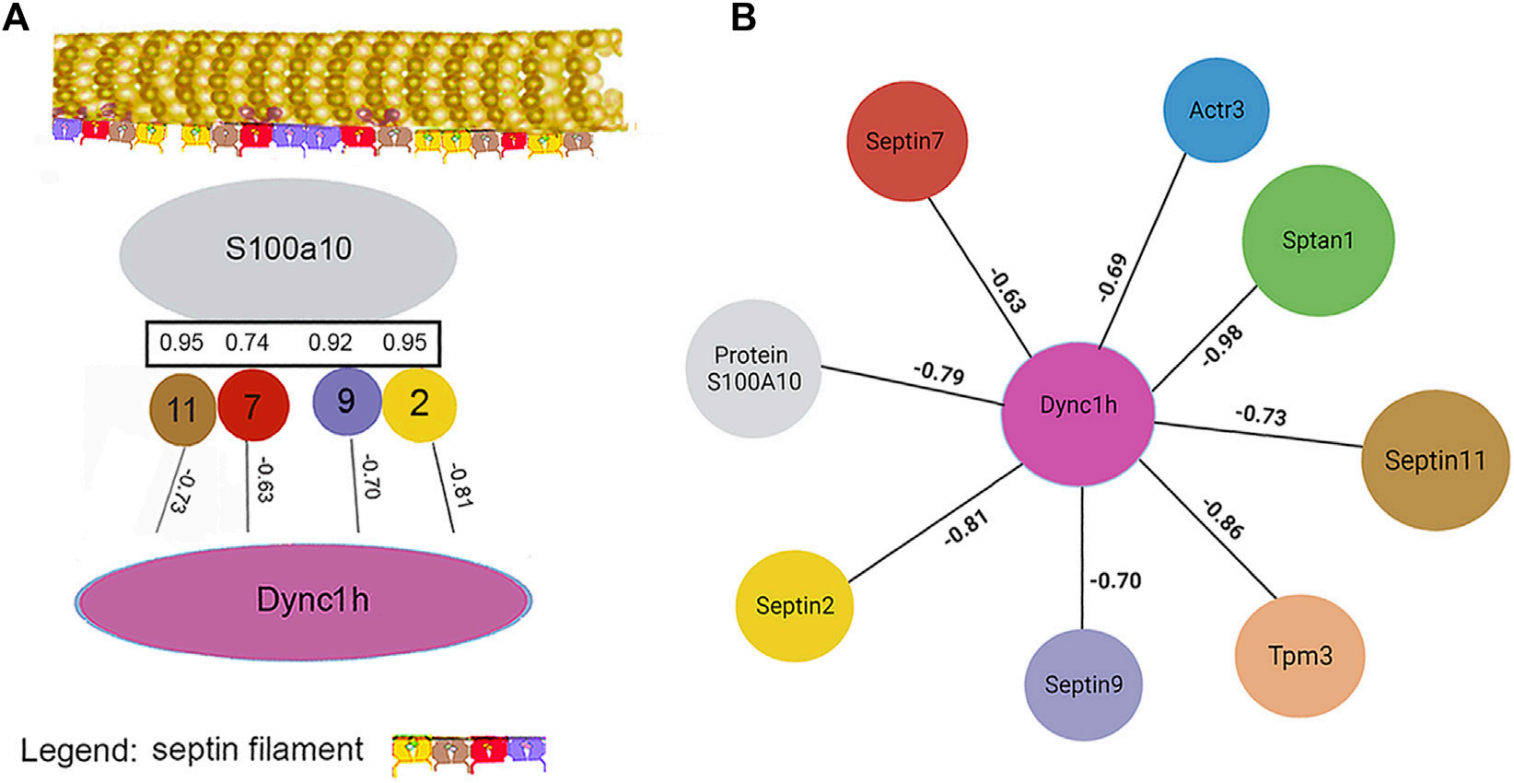
Septins cooperate with PPIN1 while avoiding Dync1h. Except for the interactions with PPIN1, correlations between septins and other proteins are mainly negative in sign, so Pearson coefficients (R) are shown. **(A)** Septins are correlated with PPIN1 core constituents, represented here solely by S100a10. Septin-11 binds β-tubulin 2a (Tubb2a), as indicated at top. Dync1h is excluded from sites that have septins. **(B)** PPIN2 is defined by a negative relationship between Dync1h and all PPIN1 proteins, represented by Sptan1 and S100A10.

Work by the Spiliotis group showed that, unlike septin-2, -6, and -7, septin-9 could regulate lysosome trafficking by affecting dynein-mediated movement. When bound to lysosomes, it recruited dynein to the membranes of these organelles (Kesisova et al., 2021), and they moved in the retrograde direction. When −2, −6, and −7 were the only septins present in experiments *in vitro*, they inhibited dynein and kinesin movement on microtubules (Suber et al., 2023). The cells of the current research lacked septin-6, but −11 is in the same subclass and is considered interchangeable with septin-6. While proteins generally showed little correlation with the abundance of α-tubulin subunits, septin-2 correlation with β-tubulin 2a (Tubb2a) was negative (R = −0.81). This was in marked contrast to the relationship of septin-11 with Tubb2a, where the correlation was high and positive (see Section 3.1.3). The remaining septins, i.e., −7 and −9, were uncorrelated with any tubulin subunit (CDs <0.15). The sign of correlation coefficients, R, between Dync1h and all septins was negative, suggesting that septins on the microtubule inhibited Dync1h from attaching to it (Figure 4A). This was consistent with results suggesting that septin-9 impedes dynein motor movement [see for review (Spiliotis and Kesisova, 2021)]. There were only a few proteins that had noticeable correlations with Dync1h (see Section 3.2)*.*

The negative correlation of septin-2 with Tubb2a suggested that it may not bind to microtubules. As a part of the whole filament, however, it may affect septin turnover on the microtubule by reducing the overall binding energy. Septins-2 and septin-9 had positive coefficients, R, with the PPIN1 of Figure 3, as discussed below (see Section 3.2). Although uncorrelated with any tubulin subunit, Dync1h was negatively correlated with all proteins of the PPIN1 pedestal, represented by S100a10 in Figure 4B. Very few proteins were potential interactors with Dync1h. As its putative binding partners were not selectively recovered with anti-keratin antibody, the results are discussed below (see Section 3.2)*.*

#### 3.1.3 Potential interactors with keratins, membranes, or adhesive specializations

Among the additional proteins that could play a structural role, e.g., components of plasma membrane, vesicles, cell-cell junctions, cell-substrate adhesive contacts, or phase-separated condensates (Table 3), catenin-β1 and 14-3-3 were known to bind keratins. Their abundance was unrelated to total keratin content in the current samples, however. Cornifin A (Sprr1a) had the highest CD with keratin abundance. In keratinocytes, cornifin is cross-linked to many other proteins by transglutaminase type I (Steinert et al., 1998). Tgm1, with the same cross-linking function, was found (Table 3). As these crosslinks were thought to reflect the extent of terminal differentiation in the cultures, cornifin was not considered relevant to the search for cross-linkers. Tgm1 was found in a Krt19 spot on two-dimensional gels and was the only putative scaffold protein of Table 3 that was both enriched by IP and found in cytoskeletal pellets. Although Anxa5 showed a CD of 0.85 with Krt17, most CDs with keratins were low. A weak value was observed for RNA-binding protein, caprin1. This protein forms phase-separated membraneless condensates in stressed cells and was found to regulate the expression of Wilms’ tumor 1-associating protein (Gao et al., 2023). Although the keratin-binding subunit of integrin, b4, was recovered, it was uncorrelated with either total keratin (Table 3) or Krt17 content. Niban2, another protein that varied in abundance with Krt17 (CD 0.88), was not known to affect the cytoskeleton, but it showed CDs of 0.87 and 0.80 with Anxa5 and Baiap2 (Table 3), which were also correlated with Krt17.

Septin-11 abundance showed little correlation with keratin or Krt17 abundance, but as a part of the septin filament, it was correlated with both the PPIN1 and the microtubule. Its CD with Tubb2a, 0.98, was the highest observed for any protein with a cytoskeletal subunit, which suggested constitutive binding. Septin-11 was also positively correlated with PPIN1 proteins (α-spectrin, Anxa2, Baiap2l, S100a10, and fascin), as were septins −2 and −9. These constituents did not appear to recruit septin-7 (see Supplementary Material S5), nor did Tubb2a. Whereas septin-2 was biased against microtubule binding, as indicated above, septin-11 was biased in favor of binding. This suggested the possibility of its forming filaments on both the PPIN1 and microtubule. Thus, switching a protein to bind to actin or microtubules may rely on recruiting septin-11. Whereas this could depend on septin-11 posttranslational modifications, only methionine loss and N-terminal acetylation [Met-loss + Acetyl (N-Term)] were found (Table 3).

#### 3.1.4 Proteins without scaffold interactions selectively recovered with antibody

Some miscellaneous proteins were analyzed. The rationale for this was that a protein with unknown localization may interact with a cross-linker or scaffold or with a keratin and thereby affect dynamic reorganization of architecture. None of these proteins showed a high correlation with keratin abundance (Table 4), however. When we computed their CDs with Krt17 abundance, adenosylhomocysteinase (Ahcy) was correlated (0.92), although no relationship between Ahcy and any cytoskeletal protein was known.

**TABLE 4:** Proteins without cytoskeletal associations selectively recovered with antibody.

Accession	Gene	Protein description	MW	R^2^	Modifications
P62193	Psmc1	26S proteasome regulatory subunit 4	49.2	0.362	
Q63347	Psmc2	26S proteasome regulatory subunit 7	48.5	0.045	
P62198	Psmc5	26S proteasome regulatory subunit 8	45.6	0.847	
P63326	Rps10	40S ribosomal protein S10	18.9	0.069	
P38983	Rpsa	40S ribosomal protein SA	32.8	0.001	
P39032	Rpl36	60S ribosomal protein L36	105.4	0.016	
P85968	Pgd	6-phosphogluconate dehydrogenase, decarboxylating	53.2	0.156	
Q9JMI1	Aacs	Acetoacetyl-CoA synthetase	87.6	0.175	
P10760	Ahcy	Adenosylhomocysteinase	47.5	0.065	
P51635	Akr1a1	Aldo-keto reductase family 1 member A1	36.5	0.040	
P15178	Dars1	Aspartate--tRNA ligase, cytoplasmic	57.1	0.129	
P16638	Acly	ATP-citrate synthase	120.6	0.723	
P22734	Comt	Catechol O-methyltransferase	29.6	0.025	
P18395	Csde1	Cold shock domain-containing protein E1	88.8	0.000	
O08651	Phgdh	D-3-phosphoglycerate dehydrogenase	56.5	0.351	Met-loss + Acetyl [N-Term]
P55053	Fabp5	Fatty acid-binding protein 5	15.1	0.009	
P05370	G6pdx	Glucose-6-phosphate 1-dehydrogenase	59.3	0.526	Met-loss + Acetyl [N-Term]
Q66H61	Qars1	Glutamine--tRNA ligase	87.6	0.364	
Q9ESH6	Glrx	Glutaredoxin-1	11.9	0.286	
E9PU28	Impdh2	Inosine-5′-monophosphate dehydrogenase 2	55.8	0.015	
P41562	Idh1	Isocitrate dehydrogenase [NADP] cytoplasmic	46.7	0.041	
P04642	Ldha	L-lactate dehydrogenase A chain	36.4	0.005	
Q62667	Mvp	Major vault protein	95.7	0.035	
Q9QVC8	Fkbp4	Peptidyl-prolyl cis-trans isomerase FKBP4	51.4	0.367	
P35704	Prdx2	Peroxiredoxin-2	21.8	0.051	
Q9EPH8	Pabpc1	Polyadenylate-binding protein 1	70.7	0.397	
P34064	Psma5	Proteasome subunit alpha type-5	26.4	0.521	
P60892	Prps1	Ribose-phosphate pyrophosphokinase 1	34.8	0.298	
Q9QZK5	Htra1	Serine protease HTRA1	51.3	0.329	
P63088	Ppp1cc	Serine/threonine-protein phosphatase PP1-gamma catalytic subunit (with Ppp1cb)	37.0	0.493	
Q5XIM9	Cct2	T-complex protein 1 subunit beta	57.4	0.183	Met-loss + Acetyl [N-Term]
Q7TPB1	Cct4	T-complex protein 1 subunit delta	58.1	0.004	
Q920J4	Txnl1	Thioredoxin-like protein 1	32.2	0.151	
Q9Z1A6	Hdlbp	Vigilin	141.5	0.002	Met-loss + Acetyl [N-Term]
Q62764	Ybx3	Y-box-binding protein 3	38.8	0.207	Met-loss + Acetyl [N-Term]
Q8K3Y6	Zc3hav1	Zinc finger CCCH-type antiviral protein 1	86.7	0.042	

### 3.2 Proteins not enriched by IP with anti-keratin antibody

**FIGURE 5:**
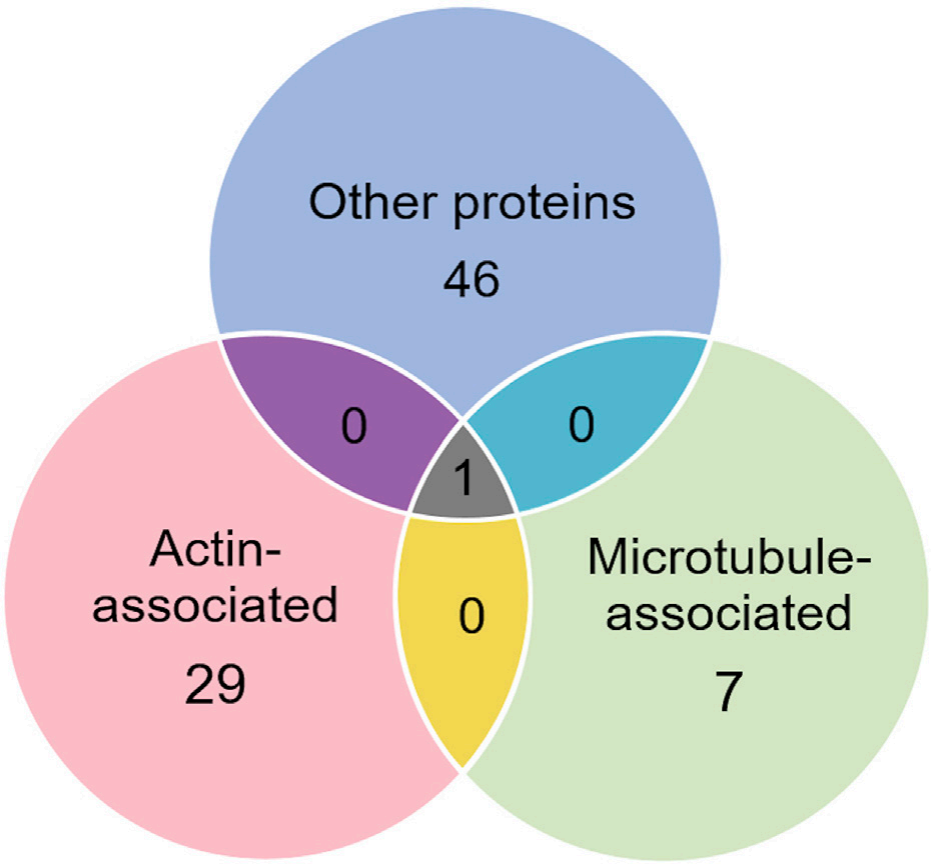
Venn diagram showing different classes of proteins that were not enriched by IP with anti-keratin. Only one, plectin, was already known to cross-link different cytoskeletal elements.

In addition to the above proteins, there were many proteins not enriched by IP that could be interacting with the cytoskeleton (Tables 5–7). The Venn diagram (Figure 5) summarizes the classes associated with different structures. Plectin was among the proteins that remained in the cytoskeletal pellet after cytoplasmic proteins were removed with TX-100. Plectin was associated with Krt5 (see Supplementary Material S6). In contrast, plectin in lysates prepared by RIPA had little correlation with overall keratin content. However, it appeared to interact with tropomyosin 4, plastin-3, and β-tubulin 2a, as judged by R values of 0.89–0.93. Other proteins that were found in cytoskeletal pellets included a tubulin subunit, Tubb5, and kinesin-1 heavy chain (Kif5b). Tubb5 was associated with a spot containing Krt14 and Krt6a, and Kif5b was associated only with the Krt14 spot. A second microtubule subunit in pellets, Tuba1c, was not well-represented in samples made by RIPA lysis. Several proteins that formed intermediate filaments or were associated with them were recovered in two-dimensional gels from cytoskeletal pellets. The scaffold, 14-3-3 protein zeta/delta, was associated with Krt19 and lamina-associated polypeptide 2 (Tmpo) with Krt5. A second lamin, prelamin A/C, was found in most of the purified keratin spots. The junction protein, plakoglobin (γ-catenin), was tightly bound to Krt5 and Krt19 (see Supplementary Material S6).

**TABLE 5:** Actin subunit and actin-associated proteins not enriched in the IP preparations.

Accession	Gene name	Protein description	MW	R^2^	Modifications
P63259	Actg1	Actin, cytoplasmic 2	41.8	0.008	Met-loss + Acetyl [N-Term]
Q9QXQ0	Actn4	Alpha-actinin 4	104.8	0.166	
Q4FZU6	Anxa8	Annexin A8	36.7	0.072	
P18484	Ap2a2	AP-2 complex subunit alpha-2	104.0	0.023	
P37397	Cnn3	Calponin-3	36.4	0.393	Met-loss + Acetyl [N-Term]
B0BNA5	Cotl1	Coactosin-like protein	15.9	0.054	
P11442	Cltc	Clathrin heavy chain 1	191.5	0.047	Met-loss + Acetyl [N-Term]
P45592	Cfl1	Cofilin-1	18.5	0.149	Met-loss + Acetyl [N-Term]; Phospho [S3]
Q7M0E3	Dstn	Destrin	18.5	0.207	Met-loss + Acetyl [N-Term]; Phospho [S3]
Q4FZY0	Efhd2	EF-hand domain-containing protein D2	26.7	0.015	
P62630	Eef1a1	Elongation factor 1-alpha 1	50.1	0.031	Acetyl [K408]
Q68FR9	Eef1d	Elongation factor 1-delta	31.3	0.006	Met-loss + Acetyl [N-Term] Phospho [S133]
Q68FR6	Eef1g	Elongation factor 1-gamma	50.0	0.762	
P05197	Eef2	Elongation factor 2	95.2	0.361	Met-loss [N-Term]
Q6AYC4	Capg	Macrophage-capping protein	38.8	0.620	Acetyl [N-Term]
O35763	Msn	Moesin	67.7	0.485	Met-loss + Acetyl [N-Term]
Q9JLT0	Myh10	Myosin 10	228.8	0.050	
P55161	Nckap1	Nck-associated protein 1	128.8	0.040	
Q63598	Pls3	Plastin- 3	70.6	0.061	Acetyl [N-Term]
***P30427***	***Plec***	Plectin*	***533.2***	***0.091***	***Met-loss [N-Term]; Phospho [S4388; S4389; S4392; Y4396; S4409]***
P62963	Pfn1	Profilin-1	14.9	0.128	Met-loss + Acetyl [N-Term] Phospho [Y7]
P05943	S100a10	Protein S100-A10	11.1	0.456	Met-loss [N-Term]
P05964	S100a6	Protein S100-A6	10.0	0.085	Met-loss + Acetyl [N-Term]
Q9QWN8	Sptbn2	Spectrin beta chain, non-erythrocytic 2	270.9	0.022	
Q9Z327	Synpo	Synaptopodin	99.9	0.319	
P61589	Rhoa	Transforming protein RhoA	21.8	0.050	
Q5XFX0	Tagln2	Transgelin-2	22.4	0.002	
P09495	Tpm4	Tropomyosin alpha-4 chain	28.5	0.260	Met-loss + Acetyl [N-Term]
Q5RJR2	Twf1	Twinfilin-1	40.1	0.001	Met-loss + Acetyl [N-Term]

Legend: ***Bold Italics*** represent proteins associated with actin, microtubules, and keratin.

**TABLE 6:** Microtubule subunits and microtubule-associated proteins not enriched by IP preparation.

Accession	Gene	Protein description	MW	R^2^	Modifications
P39052	Dnm2	Dynamin-2 (with Dmn1)	98.2	0.143	
Q04931	Ssrp1	FACT complex subunit SSRP1	80.9	0.529	Met-loss + Acetyl [N-Term]
Q2PQA9	Kif5b	Kinesin-1 heavy chain	109.5	0.003	Met-loss + Acetyl [N-Term]
Q66HR2	Mapre1	Microtubule-associated protein RP/EB family member 1	30.0	0.734	Met-loss + Acetyl [N-Term]
Q6JE36	Ndrg1	Protein NDRG1	42.9	0.004	
P13084	Npm1	Nucleophosmin	32.5	0.087	Acetyl [N-Term]; Phospho [S125]
P85108	Tubb2a	Tubulin beta-2A chain	49.9	0.036	
Q6P9T8	Tubb4b	Tubulin beta-4B chain	49.8	0.062	
P69897	Tubb5	Tubulin beta-5 chain	49.6	0.205	

**TABLE 7:** Other potential interactors with cytoskeleton that are not enriched in IP preparations.

Accession	Gene	Protein description	MW	R^2^	Modifications
P63102	Ywhaz	14-3-3 protein zeta/delta	27.8	0.480	
P61206	Arf3	ADP-ribosylation factor 3	20.6	0.106	
P61751	Arf4	ADP-ribosylation factor 4	20.4	0.091	
P84083	Arf5	ADP-ribosylation factor 5	20.5	0.060	
Q32PX2	Aimp2	Aminoacyl tRNA synthase complex-interacting multifunctional protein 2	35.4	0.008	
P35565	Canx	Calnexin	67.2	0.001	
Q07009	Capn2	Calpain-2 catalytic subunit	79.9	0.234	
P18418	Calr	Calreticulin	48.0	0.340	
Q9Z0W7	Clic4	Chloride intracellular channel protein 4	28.6	0.001	
E9PSL7	Cit	Citron rho-interacting kinase	235.2	0.040	Acetyl [K1295]
P23514	Copb1	Coatomer subunit beta	106.9	0.026	
O35142	Copb2	Coatomer subunit beta'	102.5	0.074	
Q66H80	Arcn1	Coatomer subunit delta	57.2	0.041	Met-loss [N-Term]
P97536	Cand1	Cullin-associated NEDD8-dissociated protein 1	136.3	0.236	Met-loss + Acetyl [N-Term]
O35824	Dnaja2	DnaJ homolog subfamily A member 2	45.7	0.024	
F1LP64	Trip12	E3 ubiquitin-protein ligase TRIP12	223.8	0.015	
Q80U96	Xpo1	Exportin-1	123.0	0.039	
P08753	Gnai3i	Guanine nucleotide-binding protein G(i) subunit alpha-3	40.5	0.029	
P54311	Gnb1	Guanine nucleotide-binding protein G(I)/G(S)/G(T) subunit beta-1	37.4	0.126	Met-loss + Acetyl [N-Term]
P54313	Gnb2	Guanine nucleotide-binding protein G(I)/G(S)/G(T) subunit beta-2	37.3	0.045	Met-loss + Acetyl [N-Term]
Q811S9	Gnl3	Guanine nucleotide-binding protein-like 3	60.6	0.367	
O88600	Hspa4	Heat shock 70 kDa protein	94.0	0.296	
P63018	Hspa8	Heat shock cognate 71 kDa protein	70.8	0.026	
Q66HA8	Hsph1	Heat shock protein 105 kDa	96.4	0.000	
Q6P0K8	Jup	Junction plakoglobin	81.7	0.818	Acetyl [K408]
Q62733	Tmpo	Lamina-associated polypeptide 2, isoform beta	50.2	0.589	Met-loss [N-Term]
P70615	Lmnb1	Lamin-B1	66.6	0.388	Met-loss + Acetyl [N-Term]
P43244	Matr3	Matrin-3	95.2	0.027	Met-loss [N-Term]
P21708	Mapk3	Mitogen-activated protein kinase 3	43.1	0.234	Met-loss + Acetyl [N-Term]
P21263	Nes	Nestin	208.7	0.066	
P48679	Lmna	Prelamin-A/C	74.3	0.333	Acetyl [N-Term]
Q9QZA2	Pdcd6ip	Programmed cell death 6-interacting protein	96.6	0.078	
Q4QQR9	Memo1	Protein MEMO1	33.7	0.287	
P61621	Sec61a1	Protein transport protein Sec61 subunit alpha isoform 1	52.2	0.025	
P63245	Rack1	Receptor of activated protein C kinase 1	35.1	0.167	Met-loss + Acetyl [N-Term]
Q6NYB7	Rab1A	Ras-related protein Rab-1A	22.7	0.037	
Q9WVB1	Rab6a	Ras-related protein Rab-6A	23.6	0.001	
P35281	Rab10	Ras-related protein Rab-10	22.8	0.005	
O08679	Mark2	Serine/threonine-protein kinase MARK2	80.8	0.732	
P06685	Atp1a1	Sodium/potassium-transporting ATPase subunit alpha-1	113.0	0.091	
Q63377	Atp1b3	Sodium/potassium-transporting ATPase subunit beta-3	31.8	0.132	
O08815	Slk	STE20-like serine/threonine-protein kinase	137.8	0.060	
P28480	Tcp1	T-complex protein 1 subunit alpha	60.3	0.901	Acetyl [N-Term]
Q68FQ0	Cct5	T-complex protein 1 subunit epsilon	59.5	0.117	
Q6P502	Cct3	T-complex protein 1 subunit gamma	60.6	0.061	
Q9Z269	Vapb	Vesicle-associated membrane protein-associated protein B	26.9	0.487	

The actin isoform, γ-actin, was found among the proteins not enriched by IP. It lacked any correlation with keratin content but had a CD of 0.92 with protein Ndrg1. This actin has several different functions, some of which are microtubule-associated. Of the actin-associated proteins in Table 5, the highest CD with keratin content was 0.76 for eukaryotic elongation factor 1-gamma (Eef1g). Although Eef1g had a low CD with Krt17 abundance (CD 0.37), it was still the highest value for any protein of Table 5. This surprisingly suggested a link between γ-actin and keratin structures. As two α-spectrin and two β-spectrin (Sptbn2) molecules form a tetramer, we also sought binding partners of Sptbn2. It had high CDs with RhoA (0.95) and an actin-depolymerizing factor, twinfilin (0.81).

Among the proteins associated with the cytoskeleton but not enriched by IP, we found three tubulin subunits and a microtubule-stabilizing protein, dynamin1/2 (Table 6). The latter had high CDs with heat shock protein a5 (Hspa5, 0.99), annexin A4 (Anxa4, 0.96), and actin-associated proteins, Cap1 and Wdr1 (CDs 0.92 and 0.89 respectively). This suggested an interaction with the machinery for actin severing and recycling (Ono, 2013). Among proteins of this group, there was a motor protein, Kif5b, which is implicated in anterograde transport of vimentin and keratin filaments (Robert et al., 2019), and a plus-end binding (EB) protein EB1 (Mapre1). Mapre1 showed a modest CD with overall keratin abundance (Table 6), but only 0.33 with Krt17, consistent with its not being enriched by IP.

**FIGURE 6:**
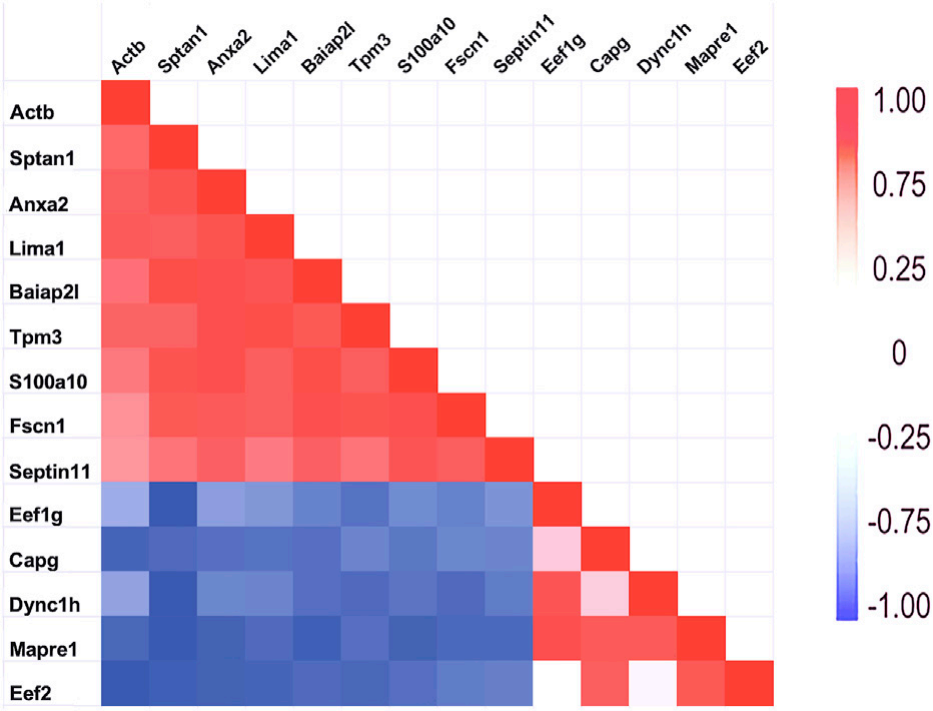
Relationship of PPIN1 to PPIN2. The heatmap represents high Pearson correlation (R) values in red and low values in blue. Dynein C heavy chain interactions with other proteins appears to depend on two binding partners. One is eukaryotic elongation factor 1 g. It is highly correlated with end-binding protein, Mapre1, as well as with Dync1h. Another eukaryotic factor, Eef2, and macrophage-capping protein (Capg) are part of PPIN2 because of strong interactions with each other and with Mapre1. As shown by the negative correlations (blue), PPIN2 excludes PPIN1 proteins. Eef2 is also negatively corelated with Actb (see Section 3.1.1).

As mentioned above, a few proteins interacted positively with dynein heavy chain (Dync1h) but were negatively correlated with PPIN1 proteins. Those interacting with Dync1h included a eukaryotic elongation factor, Eef1g (R = 0.95), and the microtubule EB protein, Mapre1 (R = 0.93). Other proteins, namely, eukaryotic elongation factor, Eef2 (R = 0.45), and macrophage-capping protein, Capg (R = 0.57) were included in the network because they had positive Pearson correlations with Mapre1 and negative correlation coefficients with all members of the PPIN1 complex (Figure 6). One example is Eef1g, which had a high negative relationship to α-spectrin abundance (R = −0.98). Another is a subunit of the heterooligomeric T-complex (Tcp-1), a complex involved in folding cytosolic proteins (Jin et al., 2019). Tcp1 showed the highest CD with overall keratin abundance of any protein recovered (Table 7). While Tcp-1 had a high correlation with dynein heavy chain (R = 0.92), its abundance was negatively correlated with the comparable values for α-spectrin (R = −0.97) and other PPIN1 proteins. There are very few members of the PPIN2 cluster, but for every member analyzed, its correlation values with PPIN1 was likely to be negative (Supplementary Material S5).

Jup, the junction plakoglobin, had a modest CD with keratin abundance (Table 7). Jup was one of only two armadillo repeat proteins recovered from most of the samples. The armadillo repeat, associated with the load-bearing function of intercellular junctions, was characteristic of two proteins detected at levels too low to be included in the analysis, namely, Diaphanous and β-catenin-like protein. A heat shock 70 protein was associated with keratins in intestinal epithelial cells (Mashukova et al., 2016). None of the heat shock proteins found in the current work were correlated with keratin or Krt17 content.

## 4 Discussion

There is a widespread consensus that cancer cells have an aberrant organization of their cytoskeletal and adhesive networks (Alibert et al., 2017; Sheetz, 2019; Xin et al., 2023). The defect may have its source in a functional node that undermines the integration of cytoskeletal networks. Although the roles of these structural elements cannot easily be deconstructed, it is possible that identifying the proteins essential for structural integration will help to address the overarching problem. To this end, we developed a database of potential interactors with cytoskeletal proteins, using an anti-Krt17 antibody in an IP protocol. Krt17 is expressed in embryonic epidermis, is inducible during wound repair, and forms heterodimers with the type II keratins, Krt6A, Krt5, and Krt8 (McGowan and Coulombe, 1998). Krt17 is not abundant in the cells studied, as Krt14 is the main acidic (type I) keratin. Strong denaturants are needed to dissociate keratin fibrils, so it was assumed that whole fibrils were present. This was supported by the finding that the main type II proteins, Krt5 and 6A, were in equal proportion to type I in the samples. As actin and tubulin subunits were also recovered, all the cytoskeletal networks were represented. The use of strong denaturants would cause the dissociation of accessory proteins from the cytoskeleton, so the PPIN networks were built up by analyzing protein-protein interactions.

The current research employed a novel approach, which combined biochemical and statistical methods to identify protein interaction networks. The rationale was that, if two proteins are persistently bound to each other, their abundances will show a constant ratio in all samples subjected to LC-MS-MS. The validity of the approach was supported by the observation of a high correlation between cornifin-A and total keratin abundance. As the main cytoskeletal proteins in the basal cells, the keratins are cross-linked to cornifin-A by transglutaminase, accounting for the correlation. The correlations of most recovered proteins with keratin content were modest, suggesting that most of these protein-protein interactions are easily dissociated. The chaperonin subunit, Tcp1, had a CD of 0.90 with keratin, but the chaperonins were known to encapsulate many proteins during folding. We found only one CD high enough to suggest constitutive binding between a recovered protein and a cytoskeletal protein, i.e., septin-11 to Tubb2a. Another protein, coatomer subunit beta’ (Copb2), was correlated with the α-tubulin 4a subunit, but there was no known relationship to microtubules.

A pool of keratin tetramers and higher order oligomers exists in cytoplasm, and it has been shown that these particles undergo retrograde movement and then merge into fibrils. In previous studies, Kölsch and coworkers showed that the particles’ movement depends on actin retrograde flow (Kölsch et al., 2009; Kölsch et al., 2010). The particles were found in a great variety of different cell types and moved continuously inward at 100–300 nm/min (Windoffer et al., 2004). Another type of saltatory movement takes the intermediate filament in a retrograde or anterograde direction (Wöll et al., 2005). This discontinuous motion was mediated by trafficking on microtubules, and both dynein and kinesin motors were implicated in filament transport (Helfand et al., 2002; Robert et al., 2019). A recent review summarizes aspects of neurofilament movement in axons and growth cones, which present fewer obstacles for visualizing fluorescent signals in living cells (Bott and Winckler, 2020).

### 4.1 Actin-associated proteins

Of the β-actin-associated proteins, only two had a CD of 0.90 or higher with Actb (Section 3.1.1) and neither protein was correlated with Krt17 or keratin abundance. Another subunit, Actg, was recovered, but only the protein, Ndrg1 (0.92), varied in abundance with Actg. Thus, high-affinity binding between any protein of our dataset and an actin subunit was not a likely basis for architectural integration.

**FIGURE 7:**
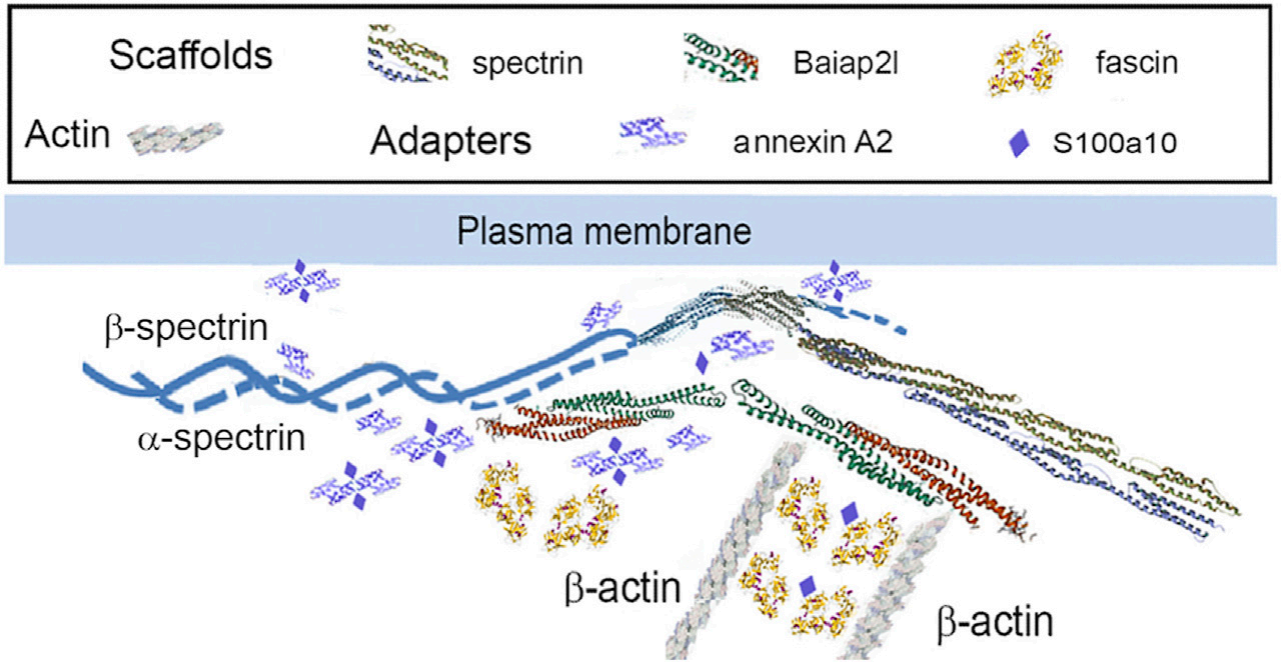
PPIN1 resembles a spectrin-IRSp53 pedestal. Fascin interacts strongly with the adapter, S100a10 and the scaffold, Baiap2l. It has weaker interactions with Macf1, Tpm3, and septin-2, and still weaker ones with Sptan1 and Anxa2. Other proteins interact with Anxa2, as judged by their CDs, but are not shown (Macf1, Myo12b, and Tpm3). Recruitment of Myo12b and Tpm3 suggests stabilization of a filament bundle, while Macf1 suggests a link to microtubules.

It appears that the Baiap2l-Sptan1 pedestal (see Section 3.1.1) may be the interface where actin-dependent movement stimulates the movement of the precursor particles. PPIN1 was made up of five core proteins, α-spectrin, Anxa2, Baiap2l, S100a10, and fascin (Figure 3). Although Sptan1 was the only node showing a correlation with keratin abundance, all had positive Pearson coefficients with keratins (Baiap2l, 0.76; Sptan1, 0.89; S100a10, 0.67; Anxa2, 0.60; Fscn1, 0.67). Moreover, one of the core proteins, Anxa2, was retained with keratins in pellets containing the more stable cytoskeletal elements. Thus, it may represent a thermodynamically stable interaction, such as would be needed to load keratin precursor particles onto actin filaments. Anxa2 is has an interesting connection to the problem of cell softening, because it is overexpressed in many types of cancer (Abe and Takeichi, 2008). While there is no known paradigm for integrating the whole set of interactions into an image, the PPIN1 core constituents can be arranged into a speculative pattern (Figure 7). Its interactions with keratin particles may rely on multiple weak interactions, acconting for the numerous constituents. In addition to Sptan and Fscn1, which are known actin-binding proteins, some of the more peripheral constituents of PPIN1 are actin accessory proteins. One subset may link the pedestal to organized actin structures, such as focal adhesions and intercellular junctions [Lima1, tropomyosin 3 (Tpm3), and myosin light chain 12b (Myl12b)]. These proteins showed low levels of variation with keratin abundance but modest correlations with actin abundance (see Section 3.1.1). Another set of constituents, represented by Macf1, may link it to microtubules.

Surprisingly, the typical binding partners of IRSp53, i.e., Eps8 (epidermal growth factor receptor kinase substrate 8), VASP (vasodilator-stimulated phosphoprotein), N-WASP (neural Wiskott-Aldrich syndrome protein), and WAVE2 (WASP-family verprolin homologous protein) (Robens et al., 2010), were absent from our samples. Also, adducin, protein 4.1, and insulin receptor tyrosine kinase substrate, constituents of the *Escherichia coli* pedestal (Ruetz et al., 2012), were not found. This may suggest that the pedestal-like complex in the respiratory airway cells is markedly different than in HeLa cells. On the other hand, Eps8 and the other IRSp53-binding proteins may bind selectively to Baiap2 rather than Baiap2l. These isoforms have considerable differences, as shown in Supplementary Material S8. As both contain an I-BAR (inverse Bin-Amphiphysin-Rvs) domain that enforces a negative curvature on a membrane by binding phosphatidylinositol-4,5-bisphosphate (Tsai et al., 2022), a mixture of Baiap2 and Baiap2l could hypothetically bind to the plasma membrane and to Krt17. Another possible means of attaching the pedestal-like complex to the membrane might rely on Lima1, also called Eplin. Lima1 has N- and C-terminal actin-binding domains and stabilizes the circumferential belt of adherens junctions in kidney and colon cells by binding α-catenin (Abe and Takeichi, 2008). However, α-catenin is absent from our samples. The Pearson coefficients, R, were 0.97 or greater for Lima1 with Myl12b, Macf1, Tpm3, and β-catenin, and all were positive in sign. While all these proteins showed at least modest positive correlations with β-actin abundance, Macf1, Myl12b, and Tpm3 were also positively correlated with Baiap2l (see Supplementary Material S5). Thus, many of the actin-binding proteins may interact with Lima1 in a cluster centered on Baiap2 (see Section 3.1.1). Lima1 appears to bind sidewise on the filament and thereby inhibit nucleation of branches by Arp2/3 (Maul et al., 2003). An alternative mechanism by which Baiap2/IRSp53 could organize sites of keratin precursors at the membrane may involve 14-3-3 binding. Baiap2 interaction with 14-3-3 proteins promotes residence of the complex at lamellipodia (Robens et al., 2010). Further work is needed to clarity the relationships among the proteins of PPIN1.

### 4.2 Microtubule-associated proteins

The results with the microtubule-associated motor protein, Dync1h, suggested that this protein participated in a different network. Again, cooperative interaction among the constituents appeared to be important, but the PPIN2 was most clearly defined by its repulsion to all constituents of PPIN1. Among the chief constituents of PPIN2 (see Section 3.1.2) was the eukaryotic elongation factor 1 g (Eef1g). It is thought to be bound to actin through its interaction with Eef1a (Cristiano, 2020), but its relationship to microtubules was not previously suspected. Nevertheless, the CD for interaction with Mapre1 is 0.95, indicating a close relationship. It is possible it is a dual-use protein (Cristiano, 2020) and participates both in protein synthesis and in trafficking of cargo of microtubules. The heatmap of Figure 6 suggests that the abundance of Mapre1, like that of Dync1h, decreased in proportion to the increased abundance of the PPIN1 network (see Section 3.1.2). Although the behavior was known to be septin-mediated from previous studies, the involvement of Eef1g is a new finding. The current work also suggests a new role for septin-11. By inference, the high positive correlation to Tuba4a, suggests it binds tubulin directly.

## 5 Conclusion

The respiratory airway cell lines are remarkably uniform in the assemblage of proteins that might interact with cytoskeletal structures. The weak binding of accessory proteins, as inferred from the correlations, suggests that cytoskeletal networks are rearranged by the concerted action of several proteins. Avid binding of proteins to cytoskeletal elements may be generally disfavored, because the architectural structures must allow different partners to be recruited as new circumstances arise. Both PPINs illustrate this principle. The α-spectrin-Baiap2l network, PPIN1, is thought to mediate the assembly of keratin precursors on fibrils. PPIN2 apparently has a role in positioning organelles in the cell. It is apparently excluded from areas occupied by PPIN1, but it is unclear what physical or functional interactions maintain the two separate areas. Moreover, it is not known whether this kind of partitioning could contribute to structural defects of cancer cells. PPIN2 includes Dync1h, which does not appear closely related to cancer, but it also includes Mapre1. This protein, also called EB1, is overexpressed in some cancers and is considered an oncogene in colorectal cancer (Stypula-Cyrus et al., 2014). Further work is required to confirm the physical relationships among the proteins of the PPINs and their patterns of dynamic rearrangement. It is vital to relate the PPINs to dynamic relationships. This is needed not only to clarify these new interactions, and most other reported interaction networks, but also to find proteins that could potentially be therapeutic targets for upper airway malignancies.

## Data Availability

The original contributions presented in the study are included in the article/Supplementary Material, further inquiries can be directed to the corresponding author. The mass spectrometry proteomics data have been deposited to the ProteomeXchange Consortium via the PRIDE partner repository with the dataset identifier PXD054199.
