# Supplementary material for: Candidate proteins interacting with cytoskeleton in cells from the basal airway epithelium in vitro
Source: Front Mol Biosci. 2024 Jul 30;11:1423503. doi: 10.3389/fmolb.2024.1423503 (PMC11319710; doi:10.3389/fmolb.2024.1423503)
Supplement: Supplementary file 1 [file DataSheet1.ZIP › Supplementary_materials/File2.docx]

| 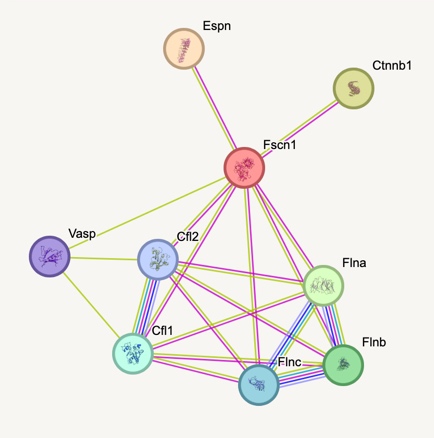 | 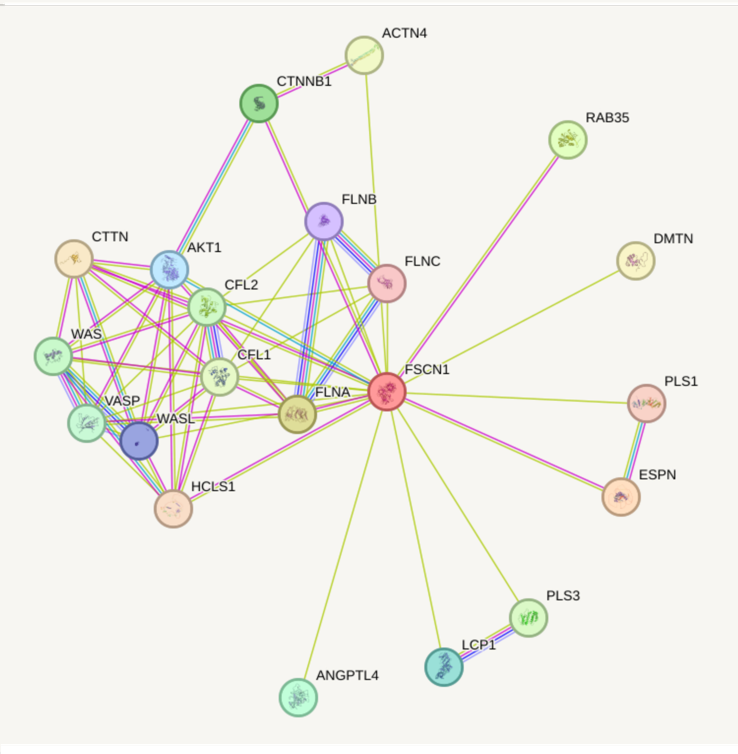 |
| --- | --- |
| (**a**) | (**b**) |

Additional File 2: STRING networks for fascin (Fscn1) show none of the PPIN1 core proteins

Frame (a) In *Mus musculus*, at confidence level 0.7, only one protein selectively recovered with the anti-keratin antibody is included, namely filamin C (Flnc). Fscn1 shows negligible correlation with Flnc. Cfl1 and Ctnnb1 were in the class of proteins not enriched by recovery with anti-keratin antibody. The Cfl1-Fscn1 correlation is negligible, whereas Ctnnb1-Fscn1 is a very weak interaction (CD 0.75). Frame (b) The corresponding *Homo sapiens* interaction network has the same proteins as above, plus a new protein, plastin 3 (Pls3), which is uncorrelated with Fscn1 abundance in the current studies.
