# Supplementary material for: Candidate proteins interacting with cytoskeleton in cells from the basal airway epithelium in vitro
Source: Front Mol Biosci. 2024 Jul 30;11:1423503. doi: 10.3389/fmolb.2024.1423503 (PMC11319710; doi:10.3389/fmolb.2024.1423503)
Supplement: Supplementary file 1 [file DataSheet1.ZIP › Supplementary_materials/File3.docx]

Additional File 3: STRING network for α-spectrin (Sptan1) in *Mus musculus*, confidence level 0.7.


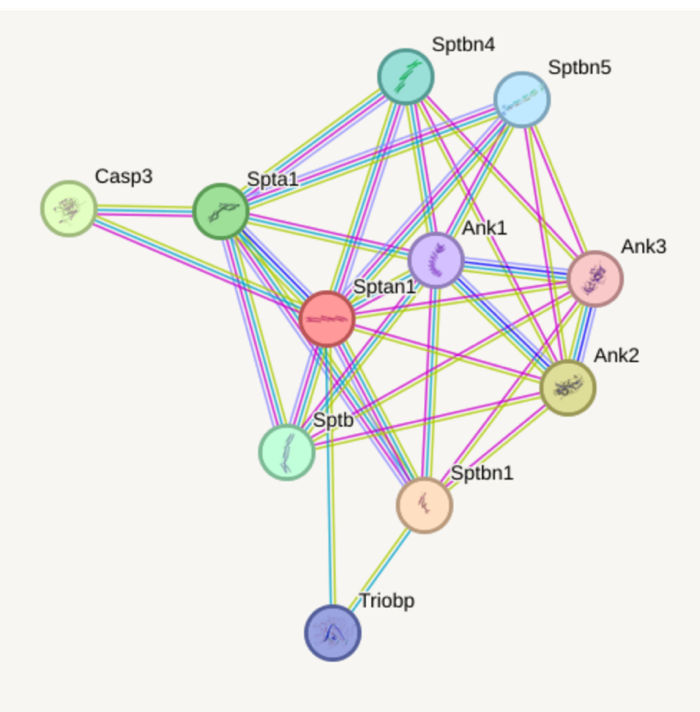


None of the spectrin interactions with keratins, Baiap2l, Annexin A2, S100a10, or fascin, referred to in the text, are represented.
