# Supplementary material for: Candidate proteins interacting with cytoskeleton in cells from the basal airway epithelium in vitro
Source: Front Mol Biosci. 2024 Jul 30;11:1423503. doi: 10.3389/fmolb.2024.1423503 (PMC11319710; doi:10.3389/fmolb.2024.1423503)
Supplement: Supplementary file 1 [file DataSheet1.ZIP › Supplementary_materials/File4.docx]

Additional File 4. STRING network for annexin A2 (Anxa2) in *Mus musculus* (confidence level 0.7)


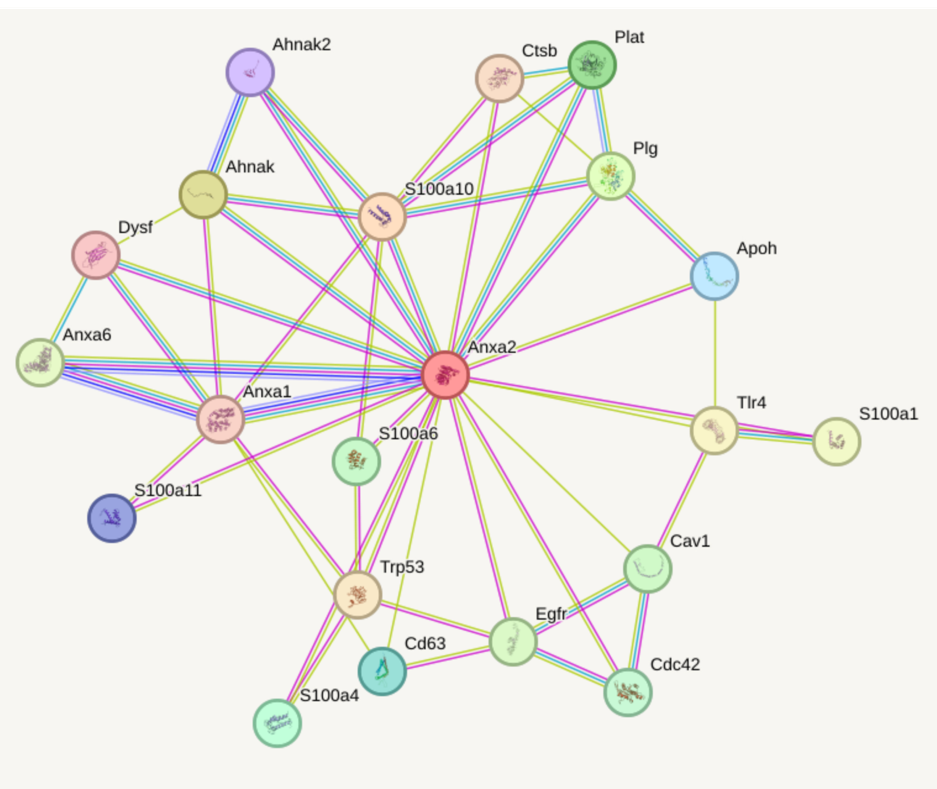


Our work suggests additional interactors, Macf1, Myl12, Lima1, and four PPIN1 constituents, that are not represented as known interactions. The STRING interactors shown (Anxa1, S100a6, S100a10, and Cdc42) have CDs of 0.06, 0.22, 0.95, and 0.00, respectively, with Anxa2 abundance (see Tables, Supplementary Material). Of the network shown, only S100a10 appeared to be bound to Anxa2.
