# Supplementary material for: Candidate proteins interacting with cytoskeleton in cells from the basal airway epithelium in vitro
Source: Front Mol Biosci. 2024 Jul 30;11:1423503. doi: 10.3389/fmolb.2024.1423503 (PMC11319710; doi:10.3389/fmolb.2024.1423503)
Supplement: Supplementary file 1 [file DataSheet1.ZIP › Supplementary_materials/File6.docx]

Additional File 6. Proteins recovered from two-dimensional gels of cytoskeletal pellets*


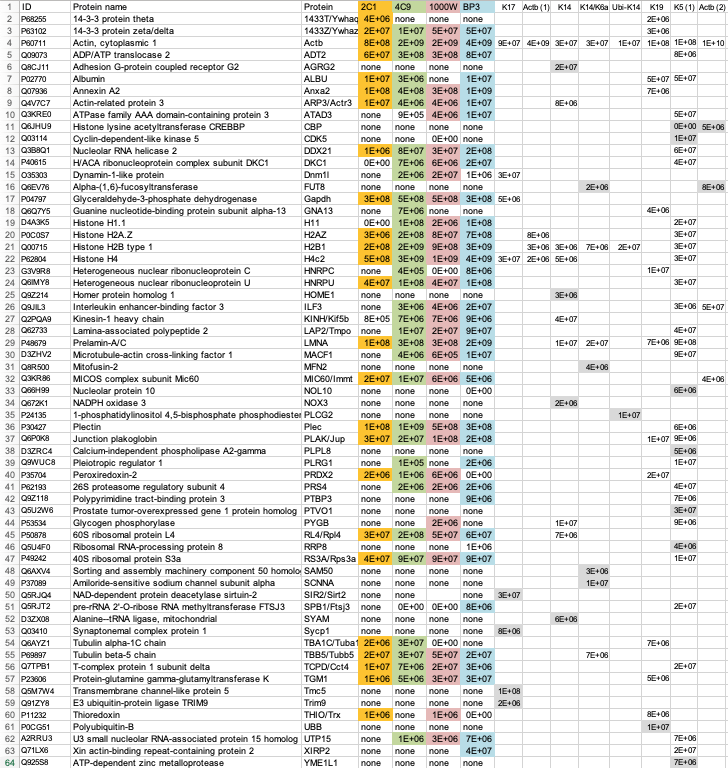


*Colors represent the four cell lines. Gray=proteins not in the dataset assembled in this research
