# Supplementary material for: Candidate proteins interacting with cytoskeleton in cells from the basal airway epithelium in vitro
Source: Front Mol Biosci. 2024 Jul 30;11:1423503. doi: 10.3389/fmolb.2024.1423503 (PMC11319710; doi:10.3389/fmolb.2024.1423503)
Supplement: Supplementary file 1 [file DataSheet1.ZIP › Supplementary_materials/File7.docx]

Additional File 7: Alignment of Baiap2l and Baiap2 curated sequences from Uniprot


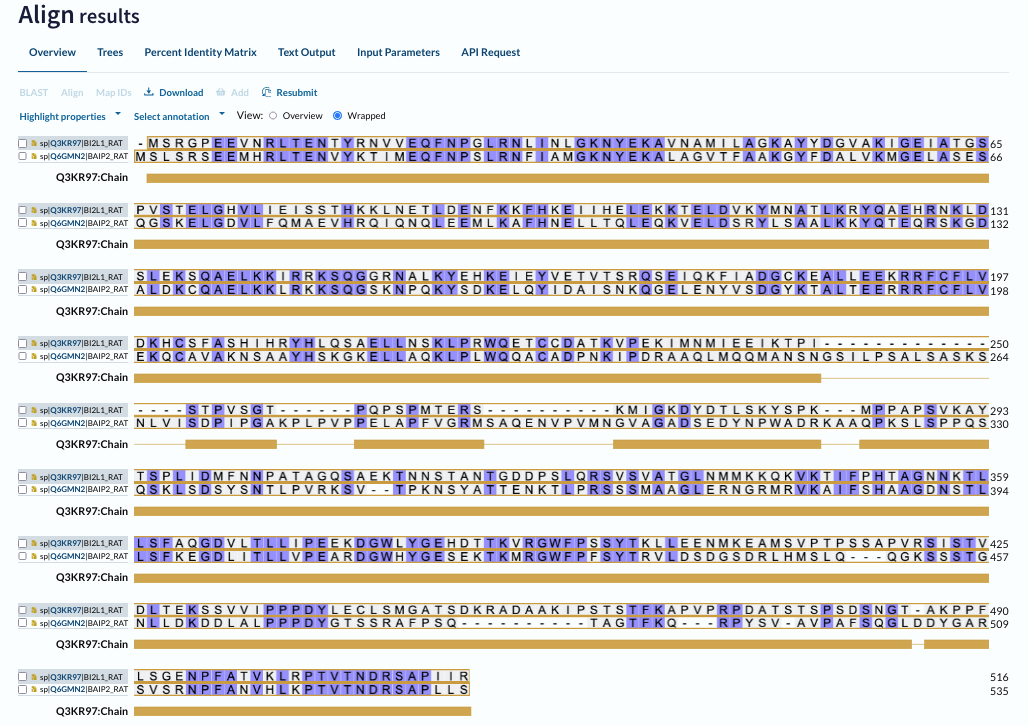


The sequences were compared using the Align service of Uniprot (www.uniprot.org/uniprot).
