# Supplementary material for: Candidate proteins interacting with cytoskeleton in cells from the basal airway epithelium in vitro
Source: Front Mol Biosci. 2024 Jul 30;11:1423503. doi: 10.3389/fmolb.2024.1423503 (PMC11319710; doi:10.3389/fmolb.2024.1423503)
Supplement: Supplementary file 1 [file DataSheet1.ZIP › Supplementary_materials/File1.docx]

Additional File 1: Antibody specificity against protein spots from SDS-polyacrylamide gels


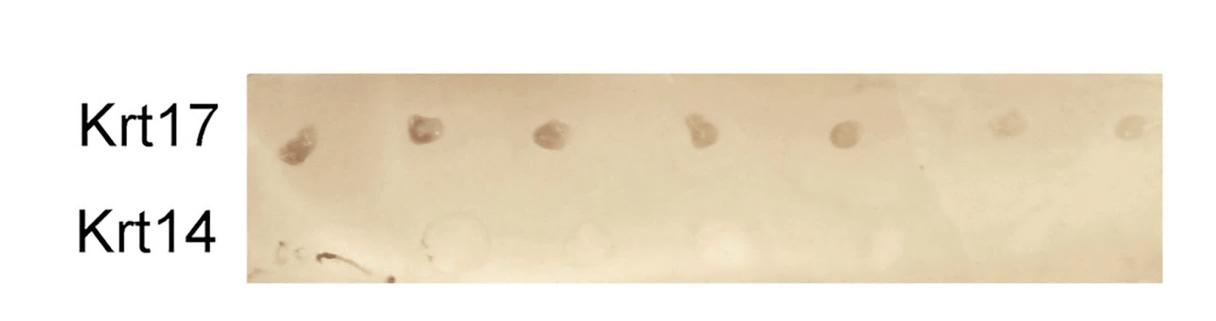


Spots representing Krt 17 and Krt 14 were excised as described in section 2, **Materials and Methods** (see *2.3 Antibody Generation and Immunoprecipitation (IP)*). Serial dilutions were made by mixing 1 part Krt17 or Krt14 to 2 parts TRIS buffer. The resulting aliquots were spotted on polyvinylidene fluoride membrane and developed as described in section 2.3.
